# First record of *Eubroncus* (Hymenoptera, Mymaridae) from China, with description of three new species

**DOI:** 10.3897/zookeys.399.6996

**Published:** 2014-04-08

**Authors:** Xiang-Xiang Jin, Cheng-De Li

**Affiliations:** 1 School of Forestry, Northeast Forestry University, Harbin, 150040, China

**Keywords:** Chalcidoidea, Mymaridae, *Eubroncus*, taxonomy, new species, China

## Abstract

The genus *Eubroncus* Yoshimoto, Kozlov & Trjapitzin is first recorded from China, and three species, *E. hani* **sp. n.**, *E. tibetanus* **sp. n.** and *E. vertexus* **sp. n.** are described as new. A key to the six described species is given, with photomicrographs to illustrate morphological characters.

## Introduction

Yoshimoto et al. (1972) established the new subfamily Eubroncinae, including two genera, *Eubroncus* and *Stomarotrum*, based on prominent mandibles that were almost as long as the head height. *Eubroncus* was erected for the type species *Eubroncus orientalis* Yoshimoto, Kozlov & Trjapitzin (male) from Malaysia, and *Stomarotrum* was erected for the type species *Stomarotrum prodigiosum* Yoshimoto, Kozlov & Trjapitzin (female) from the Russian Far East. Triapitsyn and Huber (2000) synonymized *Stomarotrum* with *Eubroncus*. Triapitsyn and Berezovskiy (2002) redescribed *Eubroncus prodigiosus* (Yoshimoto, Kozlov & Trjapitzin) and keyed males of the two known species based on color of mesosoma and whether the pronotum is divided or not. Viggiani (2003) described the male genitalial structure of *Eubroncus prodigiosus* from Japan. Lin et al. (2007) recorded an unidentified species from Australia. Hayat and Khan (2009) described the third species, *Eubroncus indicus* Hayat & Khan, from a single female from India. Here we report the genus from China and describe three new species.

## Materials and methods

Specimens were collected from Xizang Province (Tibet) and Yunnan Province (Southwest China) using yellow pan traps.

Specimens were dissected and mounted dorsally or laterally in Canada balsam on slides following the method described by Noyes (1982) and modified for the Mymaridae by Huber (1988).

Photographs were taken with a digital CCD camera attached to an Olympus BX51 compound microscope, and most measurements were made from slide-mounted specimens using an eye-piece reticle. Total body length excluding ovipositor was measured mostly with an eye-piece reticle from alcohol-preserved specimens before being dissected, but sometimes from slide-mounted specimens (meso- and metasoma, without head). All measurements are given in micrometers (μm).

Morphological terminology and abbreviations are those of Gibson (1997) and Huber (2012), as follows (with some additions):

YPT Yellow pan trap

OD Mid ocellar diameter

POD Post ocellar diameter

OOL Ocular-ocellar length

OCL Least post ocellus-occipital margin length

LOL Least ocellar length

POL Postocellar length

MOL Least mid ocellus-occipital margin length

Fln Flagellar segment

Mps Multiporous plate sensilla

Gtn Gastral tergum

Gsn Gastral sternum

Specimens studied are deposited in the following institution:

NEFU Northeast Forestry University, Harbin, China.

## Taxonomy

### Genus Eubroncus

Yoshimoto, Kozlov & Trjapitzin, 1972

http://species-id.net/wiki/Eubroncus

Eubroncus Yoshimoto, Kozlov & Trjapitzin, 1972: 879. Type species: *Eubroncus orientalis* Yoshimoto, Kozlov & Trjapitzin, 1972, by original designation.
Stomarotrum Yoshimoto, Kozlov & Trjapitzin, 1972: 879. Type species: *Stomarotrum prodigiosum* Yoshimoto, Kozlov & Trjapitzin, 1972, by original designation; synonymy by Triapitsyn and Huber 2000: 603.

#### Diagnosis.

**Figures 1–9.**
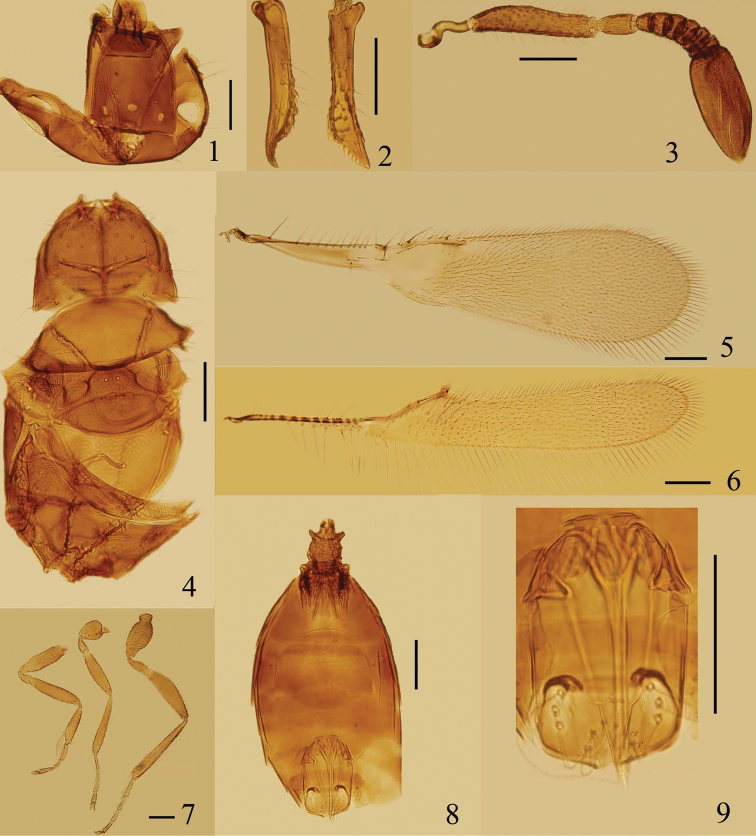
*Eubroncus hani* sp. n., holotype female: **1** head, dorsal **2** mandibles **3** antenna **4** mesosoma, dorsal **5** forewing **6** hind wing **7** legs **8** metasoma, dorsal **9** ovipositor. Scale bars = 100 μm.

**Figures 10–19.**
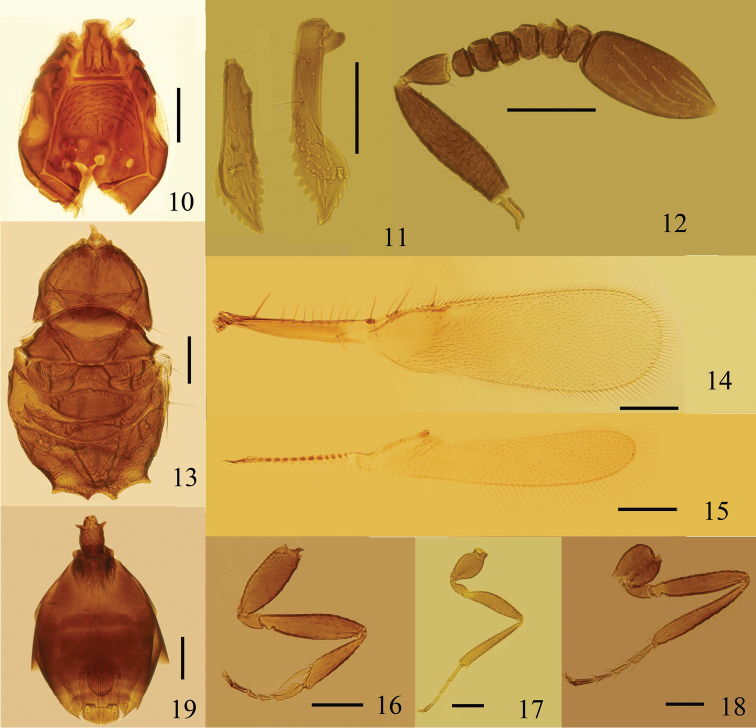
*Eubroncus tibetanus* sp. n., holotype female: **10** head, dorsal **11** mandibles **12** antenna **13** mesosoma, dorsal **14** forewing **15** hind wing **16** fore leg **17** middle leg **18** hind leg **19** metasoma, dorsal. Scale bars = 100 μm.

**Figures 20–27.**
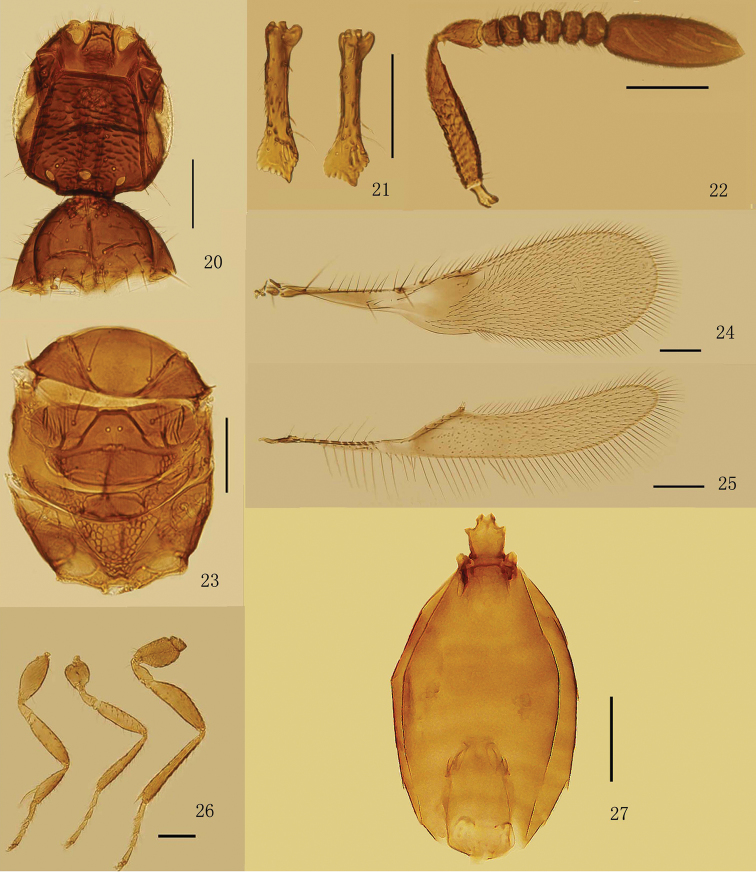
*Eubroncus vertexus* sp. n., holotype female: **20** head and pronotum, dorsal **21** mandibles **22** antenna **23** mesosoma (except pronotum) **24** forewing **25** hind wing **26** legs Paratype female: **27** metasoma. Scale bars = 100 μm.

**Figures 28–31.**
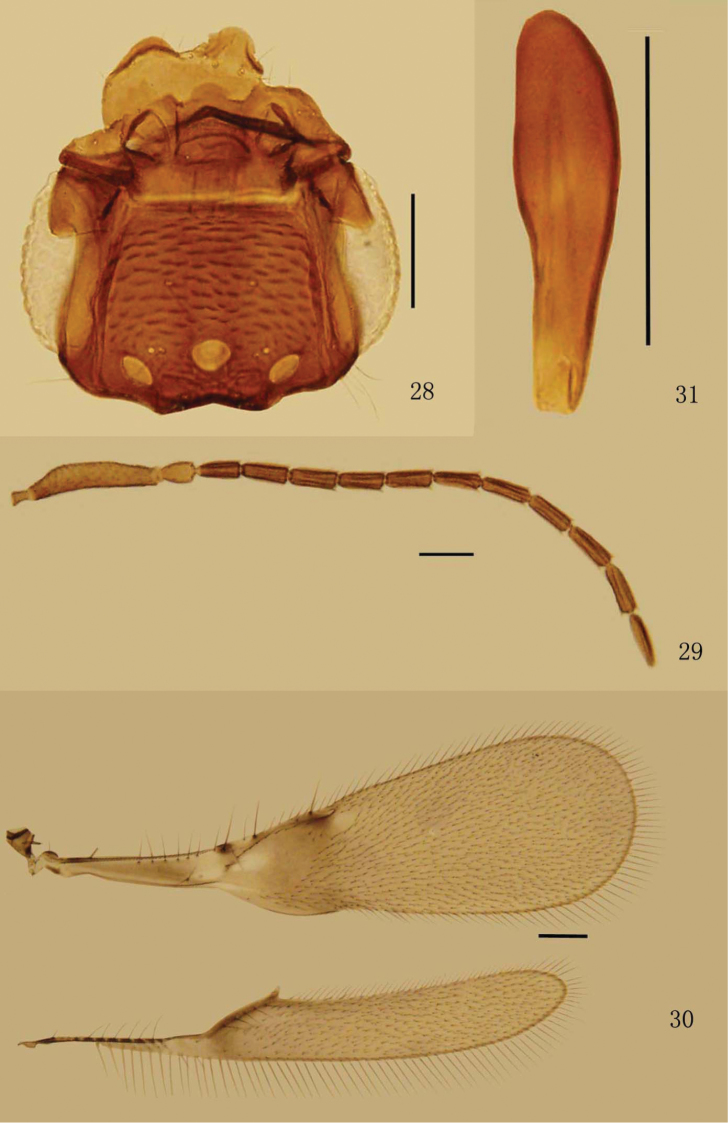
*Eubroncus vertexus* sp. n., paratype male: **28** head, dorsal **29** antenna **30** wings **31** genitalia. Scale bars = 100 μm.

Head strongly angular (or subtriangular) in lateral view. Vertex (Figs 1, 10, 20, 28) with a pair of placoid sensilla in front of post ocelli. Mandibles (Figs 2, 11, 21) not crossing medially, extremely long and narrow, with strong apical teeth and rows of denticles on ventral margin. Female antenna with funicle 6-segmented and clava 1-segmented. Pedicel distinctly longer than fl_1_, fl_1_ without mps, fl_2_–fl_6_ and clava each with numerous mps. Hind wing (Figs 6, 15, 25, 30) relatively wide with broadly rounded apex, disc begins at wing’s base, submarginal vein striped by alternating hyaline and infuscate areas. Tarsi 4-segmented. Protibial spur (Figs 7, 16, 26) comb-like. Male antennal flagellum (Fig. 29) 11-segmented.

(Note: this diagnosis applies to examined species from China because authors have not examined any other material of this genus. Hosts of all *Eubroncus* are unknown)

#### Key to species of *Eubroncus* of the world

(Note: females are not known for *orientalis*; males are not known for *indicus, hani*, and *tibetanus*)

**Table:** 

1	♀: flagellum clavate, funicle 6-segmented and clava 1-segmented (Figs 3, 12, 22)	2
−	♂: flagellum filiform, 11-segmented (Fig. 29)	6
2	Pronotum entire, without longitudinal carina medially (Yoshimoto et al. 1972, Fig. 7)	3
−	Pronotum with a faint longitudinal carina medially (Figs 4, 13, 20)	4
3	Scape about 3.0× as long as wide; Gt_1_ with prominent carinae; eye circular	*Eubroncus prodigiosus*
−	Scape about 5.5× as long as wide; Gt_1_ without prominent carinae; eye oval	*Eubroncus indicus*
4	Hind wing without a group of long setae on disc behind the distal part of marginal vein (Fig. 25); petiole with short and blunt spine-like projections anterolaterally (Fig. 27); vertex entirely covered with conspicuous scale-like sculpture (Fig. 20); ovipositor 0.87–0.90× as long as mesotibia	*Eubroncus vertexus* sp. n.
−	Hind wing with a group of notably long setae on disc behind the distal part of marginal vein (Figs 6, 15); petiole with relatively long spine-like projections anterolaterally (Figs 8, 19); vertex only partly covered with conspicuous sculpture or the sculpture inconspicuous; ovipositor 0.66–0.68× as long as mesotibia	5
5	Vertex with conspicuous scale-like sculpture in anterior half, smooth in posterior half or nearly so (Fig. 10); propodeum with distinct reticulate sculpture medially (Fig. 13); scutellum with transverse row of fovea extending to lateral margins (Fig. 13)	*Eubroncus tibetanus* sp. n.
−	Both vertex and propodeum with fine reticulate sculpture (Fig. 1); scutellum with short transverse row of fovea medially (Fig. 4)	*Eubroncus hani* sp. n.
6	Pronotum entire, without longitudinal carina medially	*Eubroncus prodigiosus*
−	Pronotum with a faint longitudinal carina medially	7
7	Forewing with a notch on basal third of posterior margin (Yoshimoto et al. 1972, Fig. 4)	*Eubroncus orientalis*
−	Forewing without a notch on basal third of posterior margin (Fig. 30)	*Eubroncus vertexus* sp. n.

### Eubroncus hani

Jin & Li sp. n.

http://zoobank.org/0EE9C19F-A59C-4D9A-A984-F2A09F13A2FA

http://species-id.net/wiki/Eubroncus_hani

Figs 1–9

#### Holotype.

♀ China, Yunnan Province, Lijiang City, Mt. Yulongxueshan, 3000m, 8–9. VII. 2012, Xiang-Xiang Jin, Hui-Lin Han, Hui Geng, Chao Zhang (NEFU), YPT.

#### Paratypes.

With same data as holotype (3♀♀, NEFU).

#### Diagnosis.

Vertex with light reticulation; pronotum with a faint longitudinal carina medially; scutellum with short transverse row of fovea medially; hind wing with a group of notably long setae on disc behind the distal part of marginal vein; petiole with relatively long spine-like projections anterolaterally.

Description. Female. Body length 1.08–1.15 mm. Head black with ocelli and mandibles brown and eyes pinkish. Antenna dark brown with apical part of radicle brown and scape yellowish-brown. Mesosoma blackish with pronotum dark brown. Wings infuscate with base of forewing dark brown, largely less infuscate behind the marginal vein and apical part of submarginal vein. Legs brown except protibial spur, trochanters, basal three tarsal segments light brown. Metasoma brown with ovipositor light brown.

Head (Fig. 1) 1.11–1.15× as long as wide. Eye subtriangular, with relatively long setae, each seta longer than the diameter of a facet. Vertex 1.1–1.3× as long as wide, with light reticulate sculpture. Ocelli in an obtuse triangle; mid ocellus oval, MOL 1.2–1.8× as long as OD; post ocellus oval, OCL approximately as long as POD; POL about 4.7–5.1× as long as OOL. Mandibles (Fig. 2) 0.68–0.78× as long as head and 1.0–1.1× as long as vertex in dorsal view. Antenna (Fig. 3) with radicle 0.2–0.4× as long as scape; scape with light reticulate sculpture, 4.0–5.4× as long as wide; pedicel 1.6–2.0× as long as wide, 2.5–2.8× as long as fl_1_; fl_2_–fl_6_ each with 2 mps; clava a little shorter than scape, 2.2–2.5× as long as wide, with 6–8 mps.

Mesosoma (Fig. 4) 1.80–2.07× as long as wide. Pronotum 0.61–0.76× as long as wide, with a faint longitudinal carina medially, each lobe with about 24–33 setae dorsally. Mesoscutum 0.41–0.47× as long as wide, and 0.72–0.83× as long as pronotum. Scutellum 0.83–0.96× as long as mesoscutum, with short transverse row of fovea medially; distance between placoid sensilla 1.4–1.6× as long as their own diameter. Propodeum 0.85–1.00× as long as mesoscutum, 1.0–1.1× as long as scutellum, with light reticulate sculpture, with one pair of tooth-like projections posterolaterally and 2–3 fine setae.

Forewing (Fig. 5) 3.86–4.10× as long as wide, longest marginal setae about 0.25–0.34× as long as greatest wing width. Beneath or on the submarginal vein with 9–12 setae. Marginal vein with 8–11 setae along anterior margin. Hind wing (Fig. 6) 7.6–8.0× as long as wide, longest marginal setae about 0.86–0.93× as long as greatest wing width, with 1 long seta and 1 short seta on marginal vein, and a group of notably long setae on disc behind the distal part of marginal vein.

Petiole (Fig. 8) 1.22–1.45× as long as wide, with relatively long spine-like projections anteriolaterally. Gaster oblong, 0.90–1.05× as long as mesosoma; Gt_1_ and Gs_1_ with numerous prominent and sclerotized carinae; ovipositor (Fig. 9) not or only slightly exserted; about 0.66× as long as mesotibia (Fig. 7).

Measurements (length/width, mm): head 0.30–0.35/0.26–0.30, scape 0.180–0.220/0.048–0.054, pedicel 0.067–0.072/0.036–0.038, fl_1_ 0.024–0.031/0.031–0.036, fl_2_ 0.024–0.034/0.038–0.043, fl_3_ 0.024–0.031/0.036–0.043, fl_4_ 0.024–0.029/0.038–0.048, fl_5_ 0.024–0.034/0.036–0.048, fl_6_ 0.024–0.036/0.045–0.055, clava 0.192–0.211/0.084–0.091, forewing 1.00–1.20/0.24–0.29, longest marginal setae 0.063–0.082, hind wing 0.94–1.10/0.12–0.14, longest marginal setae 0.102–0.125, ovipositor 0.16–0.17.

Relative measurements. OD 16–18, OCL 16–18, OOL 16–18, POL 82–84, LOL 36, POD 14–16.

#### Male.

Unknown.

#### Etymology.

The species is named for Dr. Hui-Lin Han, Northeast Forestry University, China.

### Eubroncus tibetanus

Jin & Li sp. n.

http://zoobank.org/CF35CCD0-1196-4672-A46B-7B805D804554

http://species-id.net/wiki/Eubroncus_tibetanus

Figs 10–19

#### Holotype.

♀ China, Xizang (=Tibet), Linzhi County, Pailong Village, 2000m, 22–23.IX.2011, Hui-Lin Han (NEFU), YPT.

#### Paratypes.

With same data as holotype (4♀♀, NEFU).

#### Diagnosis.

Vertex with distinct scale-like sculpture in anterior half, smooth or almost smooth in posterior half; pronotum with a faint longitudinal carina medially; hind wing with a group of notably long setae on disc behind the distal part of marginal vein; petiole with relatively long spine-like projections anterolaterally.

Description. Female. Body length 1.00–1.32 mm. Head black with ocelli and mandibles brown and eyes pinkish. Antenna dark brown with radicle yellowish-brown. Mesosoma black. Wings infuscate with base of forewing dark brown. Legs brown except protibial spur, trochanters, basal three tarsal segments light brown. Metasoma brown with apex fading to brown to yellow brown.

Head (Fig. 10) 1.1–1.2× as long as wide. Eye subtriangular, with relatively long setae, each seta distinctly longer than the diameter of a facet. Vertex 1.10–1.25× as long as wide, its posterior margin 1.6–2.0× as long as anterior margin, with conspicuous scale-like sculpture in anterior half, almost smooth in posterior half. Ocelli in an obtuse triangle; mid ocellus round, MOL approximately twice as long as OD; post ocellus oval, OCL about as long as POD; POL about 3.8–4.0× as long as OOL. Mandibles (Fig. 11) 0.7× as long as head and 1.0–1.1× as long as vertex in dorsal view. Antenna (Fig. 12) with radicle 0.25–0.30× as long as scape; scape 3.8–4.7× as long as wide; pedicel 1.6–1.8× as long as wide, 2.5–3.0× as long as fl_1_; fl_2_–fl_6_ each with 2 mps; clava slightly shorter than scape, 2.00–2.35× as long as wide, with 7 mps.

Mesosoma (Fig. 13) in dorsal view 1.6–1.8× as long as wide. Pronotum 0.6–0.8× as long as wide, with a faint longitudinal carina medially, each lobe with about 29–33 setae dorsally. Mesoscutum 0.7–0.8× as long as pronotum. Scutellum about as long as mesoscutum, with transverse row of fovea extending to lateral margins; distance between placoid sensilla about 1.6–2.2× as long as their own diameter. Propodeum with strong reticulate sculpture medially, less conspicuous laterally, about as long as mesoscutum, with one pair of tooth-like projections posterolaterally and 2–3 fine setae.

Forewing (Fig. 14) 4.0–4.2× as long as wide, longest marginal setae about 0.20–0.25× as long as greatest wing width. Beneath or on the submarginal vein with 8–13 setae. Marginal vein with 8–10 setae along anterior margin. Hind wing (Fig. 15) 7.2–7.6× as long as wide, longest marginal setae about as long as greatest wing width, with 1 long seta and 1 short seta on marginal vein, and a group of notably long setae on disc behind the distal part of marginal vein.

Petiole (Fig. 19) about 1.5× as long as wide, with relatively long spine-like projections anterolaterally. Gaster oblong, 0.86–1.05× as long as mesosoma; Gt_1_ and Gs_1_ with numerous prominent and sclerotized carinae. Ovipositor not or only slightly exserted, about 0.68× as long as mesotibia (Fig. 17).

Measurements (length/width, mm): head 0.35–0.39/0.30–0.32, scape 0.192–0.214/0.043–0.058, pedicel 0.060–0.070/0.036–0.043, fl_1_ 0.024–0.026/0.034–0.043, fl_2_ 0.024–0.034/0.036–0.048, fl_3_ 0.024–0.034/0.041–0.053, fl_4_ 0.022–0.031/0.041–0.050, fl_5_ 0.022–0.036/0.041–0.050, fl_6_ 0.024–0.036/0.048–0.055, clava 0.192–0.197/0.079–0.094, forewing 0.98–1.00/0.23–0.25, longest marginal setae 0.049–0.061, hind wing 0.90–0.95/0.12–0.14, longest marginal setae 0.122–0.129, ovipositor 0.15.

Relative measurements. OD 16, OCL 15–18, OOL 20, POL 80, LOL 32–36, POD 16.

Male. Unknown.

#### Etymology.

The specific name is derived from the name of the collection locality of the type species.

### Eubroncus vertexus

Jin & Li sp. n.

http://zoobank.org/795F2E2F-1D12-4B09-BBD4-4E4697E0235F

http://species-id.net/wiki/Eubroncus_vertexus

Figs 20 –31

#### Holotype.

♀ China, Yunnan Province, Baoshan City, Tengchong County, Laifengshan National Forest Park, 16–19.VII. 2012, Xiang-Xiang Jin, Hui-Lin Han, Hui Geng, Chao Zhang (NEFU), YPT.

#### Paratypes.

**CHINA. Yunnan Province.** Longchuan County, Zhangfeng Town, 26–27.IV.2013, Xiang-Xiang Jin, Hui-Lin Han, Guo-Hao Zu, Chao Zhang (3♀♀, NEFU), YPT; Lincang City, Yongde County, Yongkang Town, 23–24. IV.2013, Xiang-Xiang Jin, Hui-Lin Han, Guo-Hao Zu, Chao Zhang (1♀, 2♂♂, NEFU), YPT.

#### Diagnosis.

Vertex entirely covered with conspicuous scale-like sculpture; pronotum with a faint longitudinal carina medially; propodeum with strong reticulate sculpture medially, less conspicuous laterally; hind wing with 3–6 long setae and 1 short seta on marginal vein, disc uniformly setose; petiole with relatively short spine-like projections anterolaterally; ovipositor 0.87–0.90× as long as mesotibia.

Description. Female. Body length 0.9–1.1 mm. Head black with ocelli and mandibles brown and eyes pinkish. Antenna dark brown with radicle yellowish-brown. Mesosoma black with pronotum dark brown. Wings infuscate, with base of forewing under the venation dark brown, and two transparent spots, one on the behind the apical part of submarginal vein and the other on base of marginal vein. Legs brown except protibial spur, trochanters, basal three tarsal segments light brown. Metasoma brown with ovipositor light brown.

Head (Fig. 20) 1.1× as long as wide. Eye subtriangular, 1.8–2.0× as long as wide, finely setose, each seta about as long as the diameter of a facet. Vertex about as long as wide, with conspicuous scale-like sculpture entirely, its posterior margin 1.6–1.7× as long as anterior margin. Ocelli in an obtuse triangle; mid ocellus oval, MOL shorter than OD; post ocellus oval, OCL approximately a little shorter than POD; POL about 3.4–4.0× as long as OOL. Mandible (Fig. 21) 0.6× as long as head and 0.8× as long as vertex in dorsal view. Antenna (Fig. 22) with radicle 0.15–0.25× as long as scape; scape 4.5–5.3× as long as wide; pedicel 1.4–1.7× as long as wide, and 2.4–2.7× as long as fl_1_; fl_2_–fl_6_ each with 2 mps; clava shorter than scape, 2.4–2.7× as long as wide, with 8 mps.

Mesosoma (Fig. 23) 1.5–2.0× as long as wide. Pronotum (Fig. 20) 0.5× as long as wide, with a faint longitudinal carina medially, each lobe with about 18–22 setae dorsally. Propleuron with reticulate sculpture. Prepectus with strong reticulate sculpture. Mesoscutum 0.8× as long as pronotum. Scutellum about 1.0–1.1× as long as mesoscutum, with short transverse row of fovea medially, distance between placoid sensilla 1.6× as long as their own diameter. Propodeum 0.83–0.97× as long as mesoscutum, and 0.86–0.93× as long as scutellum, with strong reticulate sculpture medially, less conspicuous laterally, with one pair of tooth-like projections posterolaterally and 2–4 fine setae.

Forewing (Fig. 24) 3.55–3.85× as long as wide, longest marginal setae about 0.25× as long as greatest wing width. Beneath or on the submarginal vein with 6–8 setae. Marginal vein with 6–8 setae along anterior margin. Hind wing (Fig. 25) 7.5–8.4× as long as wide, longest marginal setae about 0.8–1.2× as long as greatest wing width, with 3–6 long setae and 1 short seta on marginal vein; disc uniformly setose, without a group of long setae behind the distal part of marginal vein.

Petiole (Fig. 27) 1.25–1.45× as long as wide, with relatively short spine-like projections anterolaterally. Gaster oblong, 0.86–1.05× as long as mesosoma; Gt_1_ and Gs_1_ with numerous prominent and sclerotized carinae. Ovipositor not or only slightly exserted; 0.87–0.90× as long as mesotibia (Fig. 26).

Measurements (length/width, mm): head 0.20–0.30/0.20–0.26, scape 0.173–0.228/0.038–0.055, pedicel 0.046–0.060/0.034–0.036, fl_1_ 0.022–0.034/0.024–0.036, fl_2_ 0.022–0.034/0.034–0.043, fl_3_ 0.019–0.034/0.036–0.043, fl_4_ 0.019–0.024/0.038–0.043, fl_5_ 0.019–0.024/0.038–0.046, fl_6_ 0.019–0.026/0.038–0.048, clava 0.156–0.206/0.050–0.091, forewing 0.98–1.00/0.19–0.26, longest marginal setae 0.055–0.063, hind wing 0.67–0.93/0.12–0.14, longest marginal setae 0.087–0.098, ovipositor 0.14–0.19.

Relative measurements. OD 12–18, OCL 6–10, OOL 16–20, POL 64–74, LOL 28–30, POD 14–18.

Male. Body length 1.1 mm. Similar to female except for normal sexually dimorphic characters and the following. Head (Fig. 28) about 0.91× as long as wide. POL about 5.4× as long as OOL. Antenna (Fig. 29) with all the flagellar segments longer than wide, each with several mps. Distance between placoid sensilla 2.0–2.4× as long as their own diameter. Forewing (Fig. 30) relatively wider than in female, 3.29–3.31× as long as wide. Hind wing (Fig. 30) 7.60–7.68× as long as wide. Genitalia (Fig. 31) simple, phallobase without parameres.

Measurements (length/width, mm): head 0.30/0.33, scape 0.228–0.235/0.048–0.058, pedicel 0.046–0.060/0.034–0.036, fl_1_ 0.070–0.084/0.036–0.041, fl_2_ 0.077–0.084/0.036–0.041, fl_3_ 0.082–0.084/0.036–0.041, fl_4_ 0.079–0.082/0.036–0.041, fl_5_ 0.079–0.084/0.036–0.041, fl_6_ 0.082–0.084/0.036–0.041, fl_7_ 0.084–0.086/0.036–0.041, fl_8_ 0.084/0.036–0.041, fl_9_ 0.086/0.036–0.041, fl_10_ 0.089–0.094/0.036–0.041, fl_11_ 0.089–0.096/0.036–0.041, forewing 1.22–1.24/0.37–0.38, hind wing 1.10–1.17/0.14–0.15, genitalia 0.12–0.14.

Relative measurements. OD 24, OCL 6, OOL 14, POL 76, LOL 30, POD 26.

#### Etymology.

The specific name refers to the vertex entirely covered with conspicuous scale-like sculpture.

## Supplementary Material

XML Treatment for Eubroncus

XML Treatment for Eubroncus hani

XML Treatment for Eubroncus tibetanus

XML Treatment for Eubroncus vertexus
